# Thrombospondin-1 protects against Aβ-induced mitochondrial fragmentation and dysfunction in hippocampal cells

**DOI:** 10.1038/s41420-017-0023-4

**Published:** 2018-02-20

**Authors:** Seokjo Kang, Jayoung Byun, Sung Min Son, Inhee Mook-Jung

**Affiliations:** Department of Biochemistry and Biomedical Sciences, College of Medicine, Seoul National University, Seoul, Korea

## Abstract

Alzheimer’s disease (AD) is often characterized by the impairment of mitochondrial function caused by excessive mitochondrial fragmentation. Thrombospondin-1 (TSP-1), which is primarily secreted from astrocytes in the central nervous system (CNS), has been suggested to play a role in synaptogenesis, spine morphology, and synaptic density of neurons. In this study, we investigate the protective role of TSP-1 in the recovery of mitochondrial morphology and function in amyloid β (Aβ)-treated mouse hippocampal neuroblastoma cells (HT22). We observe that TSP-1 inhibits Aβ-induced mitochondrial fission by maintaining phosphorylated-Drp1 (p-Drp1) levels, which results in reduced Drp1 translocation to the mitochondria. By using gabapentin, a drug that antagonizes the interaction between TSP-1 and its neuronal receptor α2δ1, we observe that α2δ1 acts as one of the target receptors for TSP-1, and blocks the reduction of the p-Drp1 to Drp1 ratio, in the presence of Aβ. Taken together, TSP-1 appears to contribute to maintaining the balance in mitochondrial dynamics and mitochondrial functions, which is crucial for neuronal cell viability. These data suggest that TSP-1 may be a potential therapeutic target for AD.

## Introduction

Alzheimer’s disease (AD) is a neurodegenerative disorder characterized by senile plaques, neurofibrillary tangles, mitochondrial dysfunction, and neuronal cell death^1^. Previous studies have reported that the amyloid β (Aβ) proteins are responsible for mitochondrial impairment during disease progression^2^. Moreover, mitochondrial accumulation of Aβ is directly associated with neuronal toxicity, which can contribute to neuronal cell death in AD^3^.

Mitochondrial function is crucial for cell survival. The mitochondria are responsible for ATP production through oxidative phosphorylation (OXPHOS), conduction of signals, and regulation of programmed cell death^4^. At the same time, mitochondria are highly dynamic organelles that undergo fission, fusion, transportation, and autophagy-mediated mitochondrial degradation. Thus, balanced mitochondrial dynamics is essential for maintaining mitochondrial function and cell survival. Mitochondrial fission/fusion is one of the major components of mitochondrial quality control^5^. Several mitochondrial fission/fusion proteins have previously been discovered. Mitofusin 1 and 2 (Mfn1 and 2), as well as optic atrophy type 1 (OPA1) induce mitochondrial fusion, while dynamin-related protein-1 (Drp1) and mitochondrial fission 1 protein (Fis1) participate in mitochondrial fission^6^. Disruption of mitochondrial morphology and subsequent mitochondrial dysfunctions have often been observed in the brains of both AD patients and experimental model animals^3,7,8^. In addition, several previous studies on AD have also reported alterations in post-translational modification (PTM) or activity of Drp1, which is a mitochondrial fission protein^9,10^.

Thrombospondins (TSPs) are large oligomeric extracellular matrix proteins that are mainly secreted from astrocytes. TSPs play a major role in synaptogenesis, cell migration, and angiogenesis^11^. TSP family members are subdivided according to their organization and domain structure. TSP-1 and TSP-2 are assembled as trimers, whereas TSP-3, TSP-4, and TSP-5 are assembled as pentamers^12^. All isoforms are expressed in the brain, however, a study using purified retinal ganglion cells demonstrated that TSP-1 and TSP-2 are especially important in facilitating excitatory synapse formation^11^. Eroglu et al.^13^ found that the synaptogenic activity of TSPs is mapped into their epidermal growth factor (EGF)-like repeat domain. In addition, the EGF-like repeats of TSPs bind to the von Willebrand factor A (VWF-A)-like domain of α2δ1, the non-pore-forming auxiliary subunit of a voltage-gated calcium channel (VGCC) highly expressed in brain^14,15^. Furthermore, α2δ1 has been reported as a neuronal receptor of TSP, which regulates excitatory synaptogenesis in the central nervous system (CNS)^14^. Thus, in terms of the synaptogenic effect of TSPs, the interaction of TSP-α2δ1 may be crucial to neuronal function although the downstream signaling of α2δ1 has not been fully identified yet. Due to the ability of TSP-1 and TSP-2 to promote the formation of new synapses, several studies have investigated the protective role of TSP-1 and TSP-2 in the context of brain injuries and neurodegenerative diseases, such as in stroke and AD, respectively. The expression of both TSP-1 and TSP-2 is significantly increased after stroke, and this upregulation of TSP-1 and 2 was mainly due to the enhanced purinergic signaling in astrocytes^16^. In addition, using TSP-1/2 double knockout mice, it has been reported that synapse formation and axonal outgrowth are largely supported by TSP-1/2 after stroke^17^. In an AD study, Son and colleagues found that the amount of TSP-1 secreted from astrocytes is decreased in Aβ-treated U373MG human astroglioma cells, the brains of AD model mice, and in human AD postmortem brains. Interestingly, the intrasubicular injection of TSP-1 into AD model mouse brains attenuated the Aβ-induced downregulation of synaptic proteins and reduction of functional synaptic activity, suggesting that TSP-1 has a protective effect on AD pathogenesis^18^.

Synaptic loss and mitochondrial damage occur during the early period of AD pathogenesis, suggesting that these prominent features of early AD are primary events for AD progression^19,20^. In addition, synaptic deficit has been known to be closely associated with mitochondrial dysfunction^21^, and these pathological characteristics of AD are correlated with cognitive impairment^22^. Many lines of evidence have suggested a close association between synaptic dysfunction and mitochondrial disruption. For instance, damaged mitochondria accumulate in neuronal cell bodies in postmortem brains of AD patients^23^. In line with this report, impaired mitochondrial trafficking and synaptic deficit due to Aβ treatment in hippocampal neurons have been reported^24,25^. Furthermore, Gillardon et al.^26^ found accumulated Aβ and downregulated energy metabolism in the synaptosomal mitochondrial fraction of AD transgenic mice, indicating that Aβ may be a crucial causative factor in mitochondrial dysfunction and the subsequent synaptic deficit and loss. However, specific mechanisms and molecules associated with this phenomenon have not been fully elucidated yet.

In this study, we present evidence of the protective role of TSP-1, in the Aβ-induced disruption of mitochondrial morphology and impairment mitochondrial functions using the HT22 mouse hippocampal neuronal cell line. Treatment of the cells with TSP-1 human recombinant protein blocked Aβ-induced excessive mitochondrial fission by maintaining the relative ratio of p-Drp1 to Drp1 protein at a level similar to that observed under normal conditions. Consistently, co-treatment with TSP-1 prevented Aβ-induced mitochondrial dysfunctions such as decreased ATP production and neuronal cell death. Furthermore, by blocking the interaction between TSP-1 and its neuronal receptor α2δ1, we also showed that α2δ1 may act as one of the target receptors of TSP-1, facilitating the protective effects of TSP-1 against Aβ-induced mitochondrial impairment in HT22 cells. Thus, our study suggests that TSP-1 may be a potential therapeutic target for AD drug development with respect to mitochondrial morphology and functions.

## Results

### TSP-1 inhibits Aβ-induced mitochondrial fission in HT22 cells

**Fig. 1:**
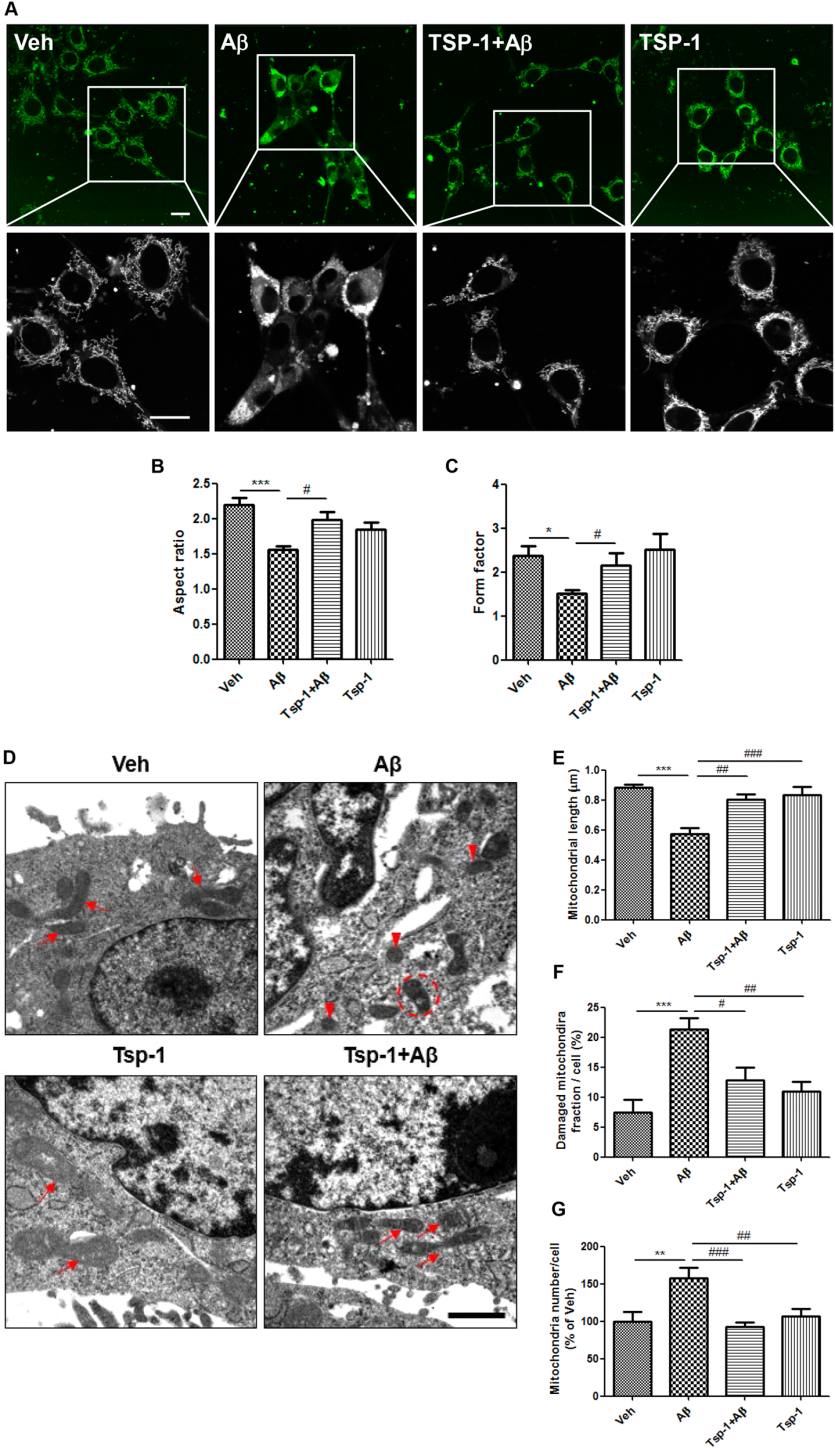
TSP-1 inhibits Aβ-induced mitochondrial fission in HT22 cells. **a** HT22 cells were stained with MitoTracker green dye to visualize mitochondrial morphology. Magnified images in lower panels are the areas marked with white lines. Scale bar, 20 μm. **b**, **c** Mitochondrial length, represented as aspect ratio and mitochondrial circular shape, represented as form factor were analyzed with the Image J program software. TSP-1 treatment significantly recovered Aβ-induced mitochondrial fragmentation. Data were obtained from at least three replicates for each group, and 100–150 cells were analyzed for each group. Data are presented as mean ± SEM. *p < 0.05, ***p < 0.001 vs. vehicle (DMSO)-treated cells; ^#^*p* < 0.05 vs. Aβ-treated cells. **d** The effect of TSP-1 on mitochondrial morphology was analyzed in EM images. Arrows indicate normal mitochondria. Arrow heads indicate fissioned mitochondria. Dashed circle indicates damaged (void) mitochondria. Scale bar, 1 μm. **e**–**g** Average mitochondrial length, number of damaged (void) mitochondria per one cell, and the total number of mitochondria per one cell were quantified. TSP-1 treatment significantly recovered mitochondrial length, decreased number of damaged mitochondria, and the total number of mitochondria per one cell. Data were obtained from at least two replicates, and more than total 10 mitochondria were analyzed for each group. Data are presented as mean ± SEM. **p < 0.01, ***p < 0.001 vs. vehicle (DMSO)-treated cells; ^#^*p* < 0.05, ^##^*p* < 0.01, ^###^*p* < 0.001 vs. Aβ-treated cells

Due to the correlation between mitochondrial dysfunction and synaptic deficit in the context of AD pathology^19–21^, we first investigated whether TSP-1 has a role not only in synaptogenesis as previously reported^11,18^ but also in mitochondrial functions during AD pathogenesis. Balanced mitochondrial dynamics between fission and fusion is one of the most important mechanisms for keeping mitochondrial quality control. Thus, we checked whether TSP-1 can affect mitochondrial morphology. HT22 cells were pretreated with human TSP-1 recombinant protein (500 ng/ml) for 1 h and then dimethylsulfoxide (DMSO) or Aβ (1 μM) was added to the medium and incubated for the next 23 h. To check mitochondrial morphology using the confocal microscope, HT22 cells were stained with MitoTracker Green FM and images were obtained from vehicle-, Aβ alone-, Aβ + TSP-1-, and TSP-1 alone-treated groups (Fig. 1a). Through Image J analysis of mitochondrial morphology using an index of aspect ratio and form factor (Fig. 1b, c), we observed that treatment with TSP-1 protein can block the Aβ-induced mitochondrial fission. To further confirm the protective role of TSP-1 in mitochondrial morphology, we analyzed the mitochondrial morphology of the same groups using electron microscopy (EM) (Fig. 1d). TSP-1 treatment completely rescued the Aβ-induced shortening of mitochondrial length, which is a characteristic of mitochondrial fission (Fig. 1e). In addition, TSP-1 also rescued the Aβ-induced increase in the damaged mitochondria fraction, which is shown as the white-colored part in the EM image due to the void content of mitochondria (Fig. 1f). Finally, TSP-1 also blocked the increase in the number of mitochondria per cell in the Aβ-treated group (Fig. 1g). These data demonstrate that TSP-1 can inhibit Aβ-induced mitochondrial fission in HT22 cells.

### TSP-1 prevents Aβ-mediated mitochondrial dysfunction in HT22 cells

**Fig. 2:**
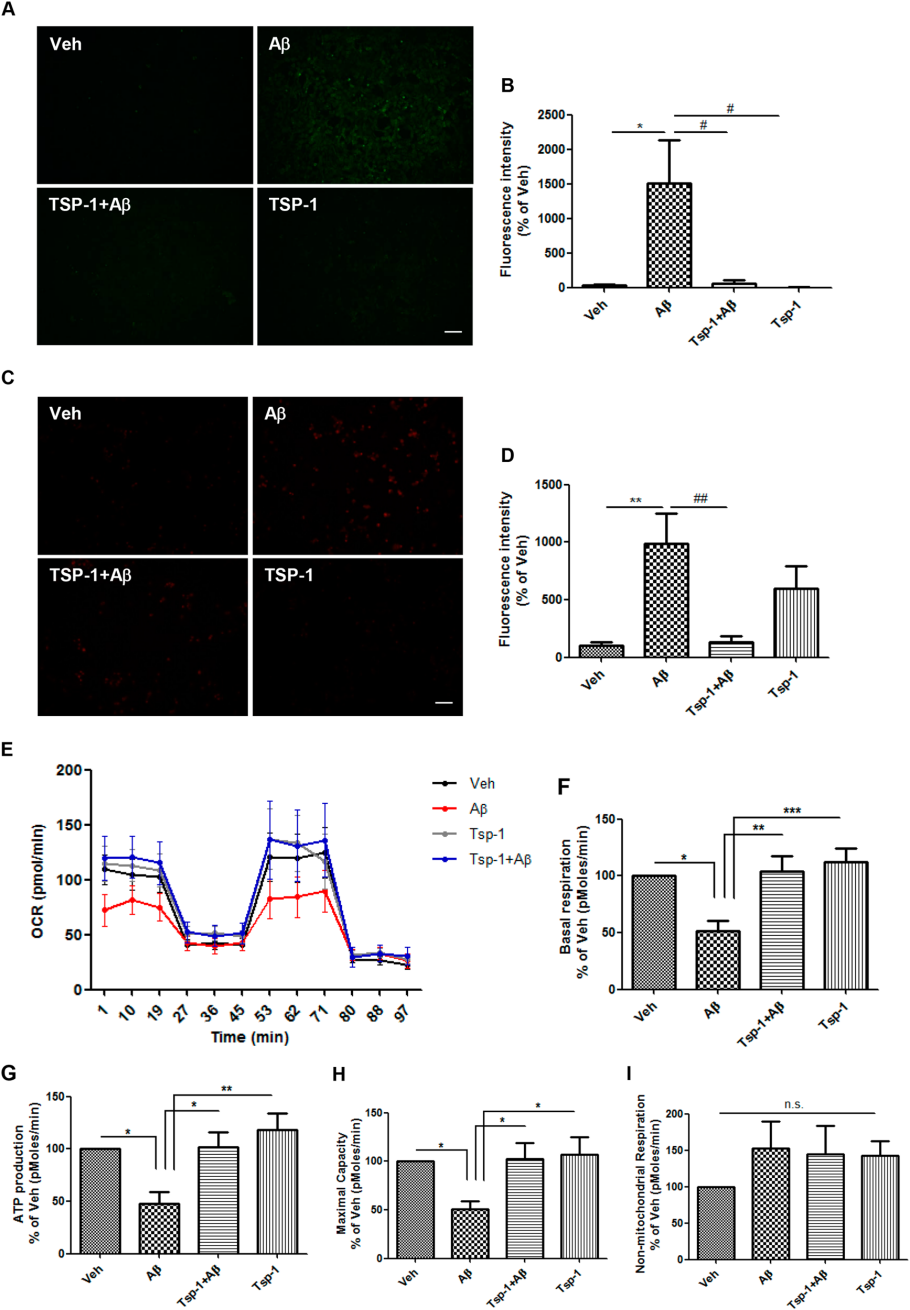
TSP-1 prevents Aβ-mediated mitochondrial dysfunction in HT22 cells. **a** HT22 cells were stained with DCFDA dye to measure cellular ROS levels. **b** TSP-1 completely blocked Aβ-mediated ROS increase. Data were obtained from at least five replicates for each group. Data are presented as mean ± SEM. *p < 0.05 vs. vehicle (DMSO)-treated cells; ^#^*p* < 0.05 vs. Aβ-treated cells. Scale bar, 50 μm. **c** MitoSOX reagent was used to detect mitochondrial specific ROS levels. **d** TSP-1 significantly inhibited Aβ-induced ROS increment. Data were obtained from at least five replicates for each group. Data are presented as mean ± SEM. **p < 0.01 vs. vehicle (DMSO)-treated cells; ^##^*p* < 0.01 vs. Aβ-treated cells. Scale bar, 50 μm. **e**–**i** Seahorse assay using XF analyzer was performed to measure oxygen consumption rate (OCR) after both Aβ only and Aβ + TSP-1 treatment. In addition, basal respiration, ATP production, the maximal capacity of the OXPHOS system, and non-mitochondrial respiration were also measured by calculating the change of the OCR value by oligomycin, FCCP, rotenone, and antimycin A administration, with a seahorse XF analyzer. Data were obtained from at least nine replicates for each group for three independent experiments. Data are presented as mean ± SEM. *p < 0.05, **p < 0.01, ***p < 0.001 vs. Aβ-treated cells

To investigate whether TSP-1 also restores Aβ-mediated mitochondrial dysfunction, TSP-1 recombinant protein was administered to Aβ-treated HT22 cells. To measure mitochondrial function, we first examined the total intracellular reactive oxygen species (ROS) and the mitochondrial ROS levels using DCFDA and mitoSOX assays, respectively (Fig. 2a, c). Co-treatment of TSP-1 and Aβ significantly blocked the increase of both total and mitochondrial ROS levels compared to Aβ-treated group (Fig. 2b, d). In addition, to measure the oxygen consumption rate (OCR) after Aβ treatment, basal respiration, ATP production, maximal capacity of the OXPHOS system, and non-mitochondrial respiration were calculated (Fig. 2e). Basal respiration, ATP production, and maximal capacity significantly recovered in the TSP-1- and TSP-1 + Aβ-treated groups compared to the Aβ only-treated group (Fig. 2f–h). However, non-mitochondrial respiration was comparable among the vehicle-, Aβ-, TSP-1 + Aβ-, and TSP-1-treated groups (Fig. 2i). Overall, these data indicate that the TSP-1 protein mitigates Aβ-induced mitochondrial dysfunction in HT22 cells.

### TSP-1 rescues neuronal cell viability against Aβ toxicity in HT22 cells and primary hippocampal neurons

**Fig. 3:**
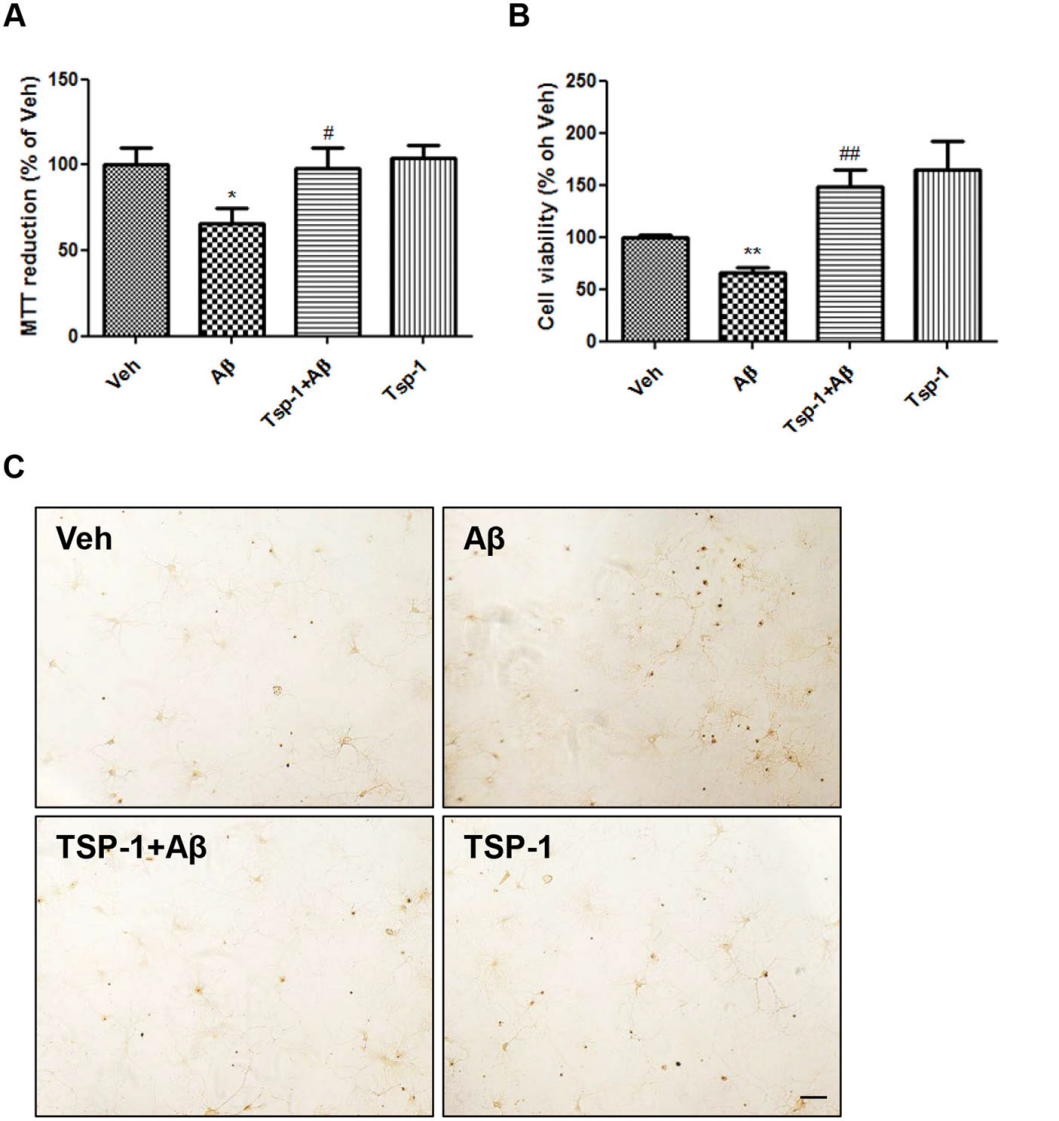
TSP-1 recovers neuronal cell viability against Aβ toxicity in HT22 cells and primary hippocampal neurons. TSP-1 plays a protective role with regards to cell viability against Aβ toxicity. Aβ-mediated cell death in HT22 cells was completely blocked by TSP-1 treatment in **a** MTT and **b** Calcein-AM assays. Data are presented as mean ± SEM. *p < 0.05, **p < 0.01 vs. vehicle (DMSO)-treated cells; ^#^*p* < 0.05, ^##^*p* < 0.01 vs. Aβ-treated cells. **c** To visually confirm the protective effect of TSP-1 on cell viability, TUNEL assay was performed in the rat primary hippocampal neurons. TSP-1 treatment considerably decreased DNA fragmentation (shown as brown or black dots) in Aβ-treated primary neurons. Scale bar, 20 μm

Since mitochondrial dysfunction caused by excessive fission leads to intrinsic apoptosis^27^, we hypothesized that TSP-1 treatment might inhibit Aβ-induced toxicity and rescue neuronal cell viability by reducing mitochondrial fission. To investigate this possibility, we performed MTT, calcein-AM, and TUNEL assays to check the cell viability of the vehicle-, Aβ-, Aβ + TSP-1-, and TSP-1-treated groups (Fig. 3a–c). Decreased cell viabilities of Aβ-treated groups were fully recovered by TSP-1 treatment in MTT, calcein-AM (HT22 cells), and TUNEL assays (primary rat hippocampal neurons). These data show that treatment with TSP-1 prevents Aβ-induced toxic effects on neuronal cell survival.

### TSP-1 restores Aβ-induced decrease of p-Drp1 to Drp1 ratio

**Fig. 4:**
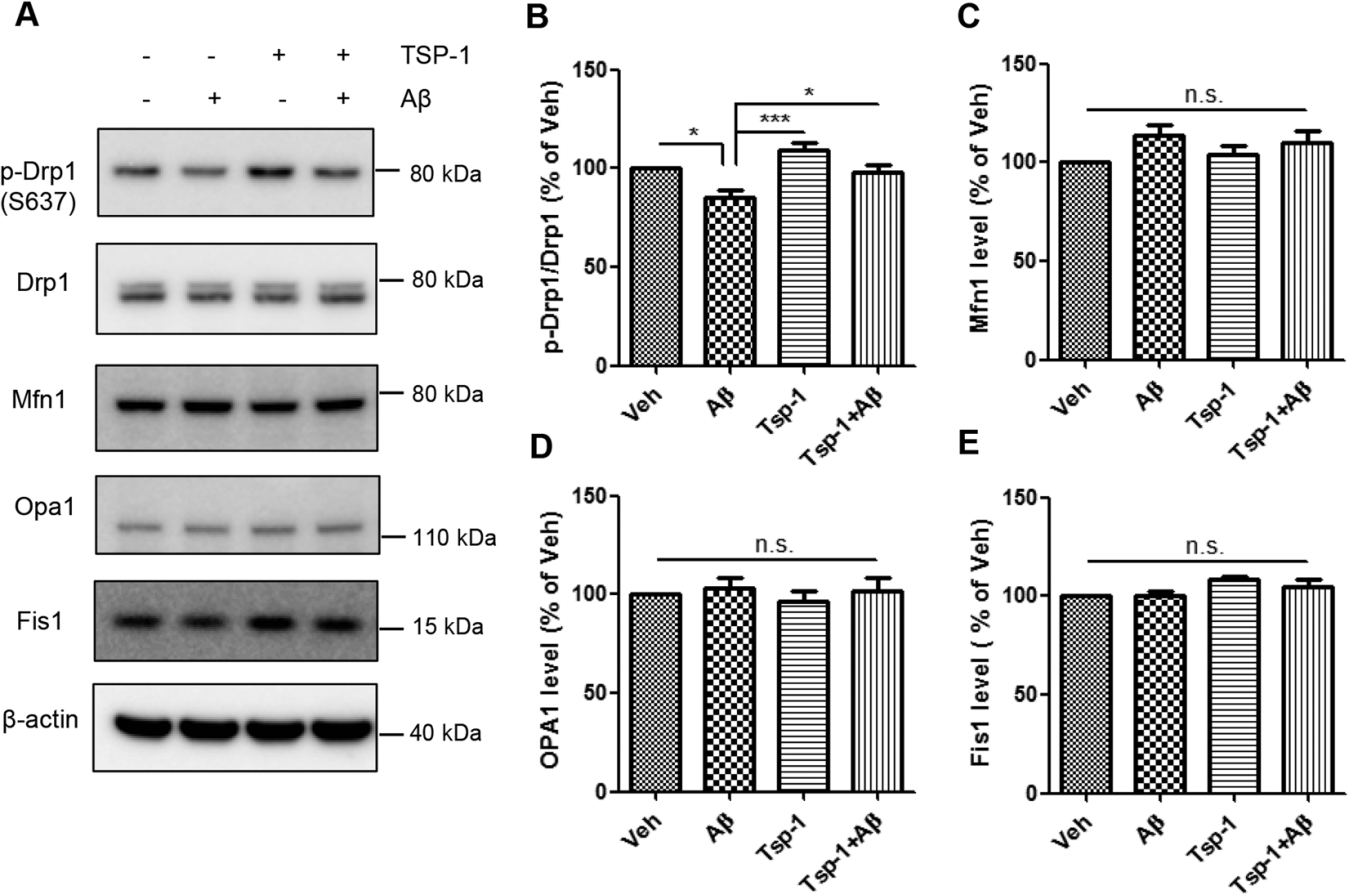
TSP-1 restores the Aβ-induced decrease of p-Drp1 to Drp1 ratio. **a** In HT22 cells, mitochondrial fission/fusion-related protein levels were determined by WB under Aβ- and/or TSP-1-treated condition. **b**–**e** Phospho-Drp1 (Ser637) to Drp1 ratio, which is important for mitochondrial dynamics was recovered by TSP-1 against Aβ toxicity while other fusion/fission proteins such as Mfn1, OPA1, and Fis1 were not changed. Data are presented as mean ± SEM. *p < 0.05, ***p < 0.001 vs. Aβ-treated cells; n.s. means statistically nonsignificant. Data were obtained from at least five replicates

One of the well-known pathological features of AD is impaired Ca^2+^ homeostasis^1^. A previous report shows that an influx of Ca^2+^ into neurons causes mitochondrial fission^28^. Since Aβ elevates the amount of intracellular Ca^2+^ level and causes mitochondrial fission, we investigated whether TSP-1 could block Aβ-mediated mitochondrial fission by directly regulating intracellular Ca^2+^ level. To identify this possibility, a fluo-4 assay was performed to measure the intracellular Ca^2+^ level in the vehicle-, Aβ-, Aβ + TSP-1-, and TSP-1-treated groups (Supplementary Figure 1a). However, TSP-1 treatment did not restore Aβ-induced intracellular Ca^2+^ elevation (Supplementary Figure 1b). Thus, we investigated whether TSP-1 could regulate mitochondrial fission proteins, especially Drp1. Drp1 is a key regulatory protein of mitochondrial fragmentation, and previous reports have demonstrated that the PTM of Drp1 is important to its activity in the fission mechanism^29^. There are various PTMs of Drp1, however, we focused on the phosphorylation of the Ser637 residue owing to its importance in both mitochondrial fission and apoptosis^30^. In addition, pSer637 Drp1 exists in the cytosol, while dephosphorylation of Ser637 Drp1 results in its translocation into the mitochondria to start the fission process^30^. To check the possibility of a regulatory role for TSP-1 on the level of p-Drp1, a western blot analysis was performed in cells from the vehicle-, Aβ-, Aβ + TSP-1-, and TSP-1-treated groups (Fig. 4a). Interestingly, the Aβ-induced reduction in the ratio of p-Drp1 to Drp1 proteins was significantly rescued by TSP-1 treatment (Fig. 4b). However, there was no alteration in levels of other fission/fusion proteins, including Mfn1, OPA1, and Fis1 upon TSP-1 treatment (Fig. 4c–e). These data suggest that TSP-1 may play a protective role in Aβ-induced mitochondrial dysfunction and neuronal cell death possibly by maintaining the ratio of p-Drp1 to Drp1 proteins.

### α2δ1 receptor is important to mediate the regulating effect of TSP-1 for the ratio of p-Drp1 to Drp1

**Fig. 5:**
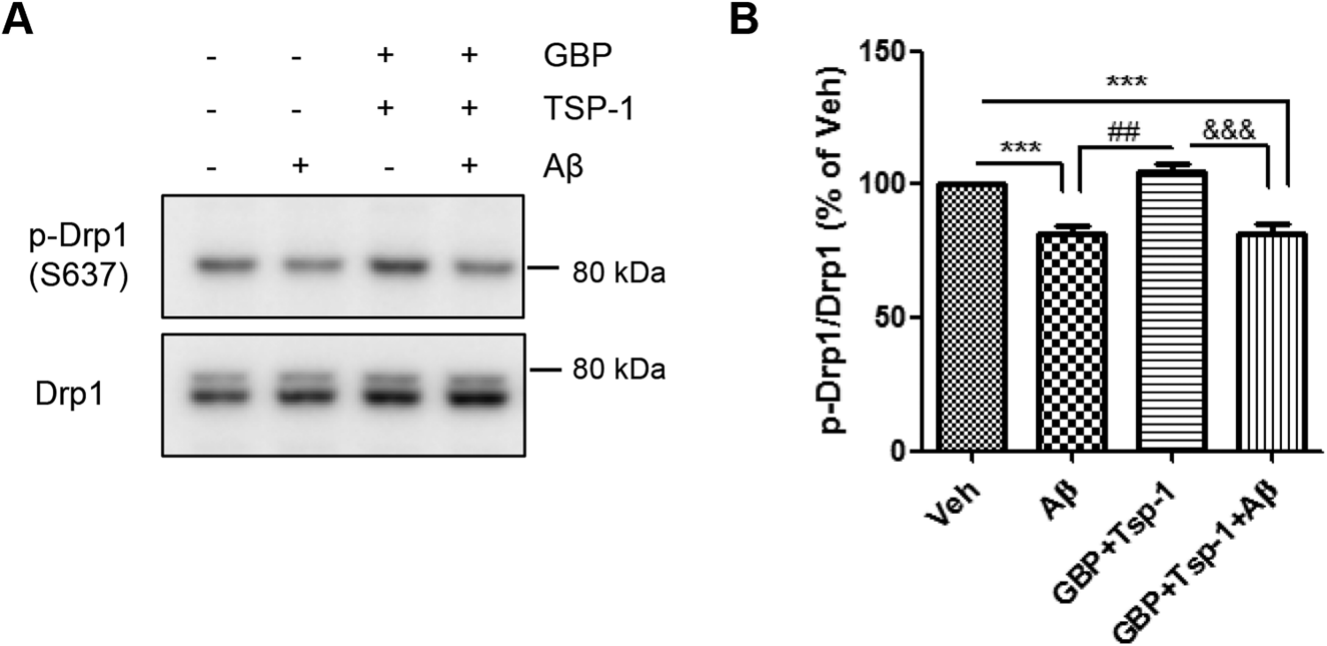
α2δ1 receptor is important to mediate the regulating effect of TSP-1 for the ratio of pDrp-1 to Drp1. **a**, **b** Gabapentin (100 μM), a blocker for TSP-1-α2δ1 interaction, suppressed the ability of TSP-1 to maintain p-Drp1 levels. Data are presented as mean ± SEM. ***p < 0.001 vs. Veh (DMSO)-treated cells; ^##^*p* < 0.01 vs. Aβ-treated cells; ^&&&^*p* < 0.001 vs. GBP + TSP-1-treated cells; n.s. means statistically nonsignificant. Data were obtained from at least five replicates

Next, we investigated the possible protective mechanism of TSP-1 on Aβ-induced mitochondrial dysfunction and reduction of neuronal cell viability. Several receptors for TSP-1 have been reported including neuroligin-1 (NL1), CD47, and α2δ1^31^. Out of these, α2δ1 is a neuronal receptor of TSP regulating excitatory synaptogenesis in the CNS^14^. Furthermore, we have previously reported that TSP-1 mitigates Aβ-induced synaptic dysfunction and reduction of synaptic proteins via the α2δ1 receptor on neurons^18^. Thus, the interaction of TSP-1-α2δ1 may be crucial to neuronal cell viability, which is directly affected by mitochondrial function, although the downstream signaling of α2δ1 has not been fully identified yet. Therefore, we examined whether the α2δ1 receptor, which is highly expressed in HT22 cells (Supplementary Figure 2), is responsible for the regulatory effect of TSP-1 on the level of p-Drp1. HT22 cells were treated with gabapentin, an analog of γ-aminobutyric acid and a known blocker of the TSP-1-α2δ1 interaction, to identify the role of the α2δ1 receptor in the action of TSP-1. Interestingly, TSP-1 treatment did not restore the Aβ-induced reduction of the ratio of p-Drp1 to Drp1 in the gabapentin pretreated group (Fig. 5a, b), suggesting that the effect of TSP-1 on the regulation of the ratio of p-Drp1 to Drp1 is through the α2δ1 receptor. Together, these data indicate that TSP-1 attenuates mitochondrial fission, mitochondrial dysfunction, and reduced neuronal cell viability caused by Aβ toxicity mainly through the α2δ1 receptor in HT22 cells.

## Discussion

In this study, we found that TSP-1 has a protective role in Aβ-induced mitochondrial fission, mitochondrial dysfunction, and neuronal cell death (Supplementary Figure 3). As a mechanistic link, we identified that TSP-1 maintains the level of p-Drp1 (Ser637), which on the molecular level is important for maintaining mitochondrial dynamics. By blocking the reduction of the p-Drp1 to Drp1 ratio in the presence of Aβ, TSP-1 contributes to maintaining mitochondrial dynamics and mitochondrial functions, which is crucial for neuronal cell viability. Finally, we observed that the protective role of TSP-1 mitigating Aβ-induced mitochondrial dysfunction may be mediated by the neuronal TSP-1 receptor, α2δ1.

Previous reports show that Aβ induces synaptic dysfunction and mitochondrial impairment^19,20^. In addition, it is known that synaptic deficit is closely associated with mitochondrial dysfunction^21^. For example, damaged mitochondria are accumulated in the neuronal cell body of AD patients’ postmortem brains^23^. In line with this report, impaired mitochondrial trafficking and synaptic deficit due to Aβ treatment have been identified in hippocampal neurons^25^. Furthermore, there is a report showing accumulated Aβ and decreased energy metabolism in the synaptosomal mitochondrial fraction of AD transgenic mice, indicating that Aβ may be a crucial causative factor in mitochondrial dysfunction and subsequent synaptic deficit and loss^26^.

TSPs are typical oligomeric matrix proteins and are primarily secreted from astrocytes to neurons in CNS. The TSP family members are subdivided according to their organization and domain structure. TSP-1 and TSP-2 proteins form trimers, whereas TSP-3, TSP-4, and TSP-5 proteins form pentamers^12^. TSPs play main roles in synaptogenesis, cell migration, and angiogenesis^11^. Recently, there have been many reports focusing on the synaptogenic activity of TSP-1. Son et al.^18^ showed that among the isoforms, only TSP-1 was downregulated in AD brains along with synaptic deficit. In addition, recovery of the normal level of TSP-1 by treatment with recombinant TSP-1 protein could attenuate Aβ-induced dendritic spine loss and synaptic dysfunction in vitro and in vivo, implying a possible role for TSP-1 in AD therapeutics^18^. However, the effect of TSP-1 in mitochondrial morphology and function that are critical for AD pathological mechanisms, have not been investigated yet. In this study, we found that TSP-1 inhibits Aβ-induced disruption of mitochondrial morphology and dysfunction, by maintaining the p-Drp1 (Ser637) to Drp1 ratio at a level similar to that observed in the normal control group via the α2δ1 neuronal TSP-1 receptor. Together with the previous results from the study of Son et al. study^18^, these data indicate that TSP-1 is a potential therapeutic candidate for AD not only because of its protective role in synaptic deficit but also because of its role in balancing mitochondrial dynamics and maintaining mitochondrial functions.

Not surprisingly, mitochondrial function is important for cell viability owing to its various roles, including ATP production through OXPHOS, conduction of signals via second messengers, and regulation of intrinsic apoptosis^4^. In addition, mitochondria are highly dynamic organelles that undergo fragmentation, elongation, trafficking for ATP supply, and autophagy-mediated mitochondrial degradation. Thus, balanced mitochondrial dynamics is crucial for maintaining mitochondrial functions. Mitochondrial fission/fusion is one of the major factors controlling mitochondrial quality^5^. Increasing evidence has revealed that Aβ increases mitochondrial fission, disturbs axonal transport, and increases synaptic degeneration, all of which are mediated by mechanisms related to the Drp1 protein^10^. Moreover, a recent study has revealed that inhibiting the activity of Drp1 ameliorates synaptic depression, Aβ deposition, and cognitive impairment in AD mice^32^. Drp1 is a key regulatory protein of mitochondrial fission, and PTM of Drp1 is important to its GTPase activity^29^. Within the diverse PTMs of Drp1, we were interested in phosphorylation of Ser637 residue by reason of its importance in both mitochondrial fission and apoptosis^30^. Besides, pSer637 Drp1 exists in the cytosol, while the dephosphorylation of Ser637 leads to the translocation of Drp1 into the mitochondria to start mitochondrial fragmentation^30^. To clarify our hypothesis about the regulatory role of TSP-1 in maintaining the ratio of p-Drp1 to Drp1 in detail, we performed a western blot assay. Surprisingly, Aβ-induced reduction in the p-Drp1 to Drp1 ratio was significantly recovered in the TSP-1-treated group in HT22 cells (Fig. 4).

What is the mechanism underlying the regulation of the p-Drp1 to Drp1 ratio by TSP-1? To solve this question, we paid attention to its receptors. TSP-1 has several receptors, including CD47, NL1, and α2δ1^31^. Owing to the known function of the TSP-1-α2δ1 interaction in the recovery of spinal loss and synaptic deficit in the AD condition^18^, as well as in synaptogenesis in the CNS^14^, we postulated that the neuronal α2δ1 receptor may be one of the most important receptors of TSP-1 in AD. To investigate whether TSP-1 acts through the α2δ1 receptor to exert its protective role against Aβ toxicity in mitochondria, we treated HT22 cells with gabapentin, a commonly prescribed anti-epileptic and anti-neuropathic medication. Gabapentin completely blocked the ability of TSP-1 to maintain the level of p-Drp1 (Ser637), which resulted in mitochondrial fission, mitochondrial dysfunction, and reduced cell viability in the presence of Aβ (Fig. 5). The α2δ1 receptor is a subunit of a VGCC, thus, the blocking effect of gabapentin against TSP-1 may be as a result of the functional modulation of the α2δ1 receptor as a calcium channel component. Gabapentin has a high affinity for the α2δ1 receptor^33^ but has been known to block the TSP-1-α2δ1 interaction without changing calcium channel expression, function, and kinetics^14^. Furthermore, the fact that α2δ1 has the VWF-A-like domain^14,15^ suggests that α2δ1 can interact with various types of protein ligands affecting a wide variety of signaling pathways independent of calcium signaling. Our data also showed that treatment of HT22 cells with TSP-1 did not rescue Aβ-induced intracellular Ca^2+^ increment, implying that the effect of TSP-1 in AD might not be dependent on the modulation of intracellular Ca^2+^ levels (Supplementary Figure 2). In addition, Garcia et al.^34^ reported a novel role for α2δ1 in myoblast attachment and extracellular signaling, which are not relevant to the calcium signaling pathway. Furthermore, Eroglu et al.^14^ also reported the calcium channel-independent synaptogenic effect of the TSP-1-α2δ1 interaction in neuronal cells. Therefore, based on our experimental data, it is highly possible that the TSP-1-α2δ1 interaction in our study may exert a protective role against Aβ toxicity via a calcium channel-independent mechanism, by affecting the regulation of p-Drp1 (Ser637) levels. We think that there may be downstream molecules that regulate the p-Drp1 to Drp1 ratio under the TSP-1-α2δ1 interaction. Further studies to determine the molecule responsible for regulating this process is required.

Thus, TSP-1 may be a potential therapeutic target in treating AD pathology by inhibiting mitochondrial dysfunction and the disruption of mitochondrial morphology. Despite the fact that there are 10 times more glial cells than neurons^35^, there have not been many studies investigating the roles of these cell types in the CNS and in neurodegenerative diseases. Since glial cells secrete many types of growth factors, neurotrophic factors, and cytokines, their role in the CNS environment appear to be critical for neuronal survival. Thus, further studies of secretory molecules, such as TSP-1, from glial cells are required to identify clinically effective therapeutics in many CNS disorders by improving the general CNS environment in which the neuron can maintain cellular viability consistently.

In conclusion, this study provides evidence that TSP-1 plays a protective role in mitochondrial dynamics, mitochondrial function, and neuronal cell viability through its neuronal receptor α2δ1 (Supplementary Figure 3). Although the precise mechanism of the TSP-1-α2δ1 interaction affecting mitochondrial dynamics is not well understood, future research focusing on the protective role of TSP-1 may reveal a potential therapeutic target for AD treatment.

## Materials and methods

### Cell cultures and drug treatment

An immortalized mouse hippocampal neuronal cell line, HT22, and rat hippocampal primary neurons were used in this research. HT22 cells were cultured in Dulbecco’s modified Eagle’s medium with 10% fetal bovine serum, and 0.1 mg/ml of penicillin and streptomycin (P/S; Sigma-Aldrich, St. Louis, MO, USA) at 37 °C, under 5% CO_2_ air. HT22 cells were counted and seeded onto 6-well plates (1.0 × 10^5^ cells/well), 12-well plates (3.0 × 10^4^ cells/well), and seahorse assay plates (1.5 × 10^4^ cells/well). Primary hippocampal neurons were cultured from Sprague-Dawley rat embryos, as previously described^18^. The neurobasal medium with B27 supplement (Invitrogen, Carlsbad, CA, USA), l-glutamine (0.5 mM), and 0.1 mg/ml P/S (Sigma-Aldrich) was used to culture primary neurons and changed every 2 days. The experiments were executed in cultures at 21 days in vitro. Cells were grown to 70% confluency and treated solely with 500 ng/ml of human recombinant TSP-1 (R&D System, Minneapolis, MN) for 24 h, or pretreated for 1 h and then co-treated with Aβ (1 μM; American Peptide) for the remaining 23 h of incubation. Other drugs used in this study were gabapentin (100 μM; Sigma-Aldrich), oligomycin (2 μM; Seahorse Bioscience, Billerica, MA), carbonyl cyanide 4-(trifluoromethoxy) phenylhydrazone (FCCP) (2 μM; Seahorse Bioscience), rotenone (0.5 μM; Seahorse Bioscience), and antimycin A (0.5 μM; Seahorse Bioscience).

### Western blot analysis

Cells were analyzed by western blotting using 4–12% SDS gradient gels (Invitrogen). The antibodies for the western blot analysis were as follows: anti-Drp1 (1:2000; Santa Cruz); anti-p-Drp1 (S637) (1:2000, Cell Signaling Technology, Beverly, MA, USA); anti-Mfn1 (1:1500; Santa Cruz); anti-OPA1 (1:500; Santa Cruz); anti-Fis1 (1:1000; Santa Cruz); anti-α2δ1 (1:1000, Santa Cruz); anti-β-actin (1:2000; Sigma-Aldrich); and anti-GAPDH (1:4000; Sigma-Aldrich). Immunoreactivity was analyzed by chemiluminescence (GE Healthcare, Piscataway, NJ, USA). The chemiluminescence signal was observed with a digital image analyzer (LAS-3000; Fuji Film Inc., Tokyo, Japan).

### Mitochondria morphology analysis

Mitochondrial morphology was investigated using MitoTracker Green FM (Thermo Fischer Scientific, Hudson, NH, USA) in drug-treated HT22 cells. Briefly, cells were pretreated with 500 ng/ml TSP-1 recombinant protein for 1 h and 1 μM Aβ or DMSO was additionally administered to the cells for next 23 h. Next, cells were stained with 100 nM MitoTracker Green FM for 20 min to visualize mitochondrial morphology and then washed two times with Opti-MEM. Live-cell imaging was taken using the confocal laser scanning microscope (FV10i-w, Olympus, Tokyo, Japan) and analyzed using the Image J program (NIH, Bethesda, MD, USA). To quantify the change in mitochondrial morphology in detail, the cells were also observed using EM as previously described^36^. HT22 cells were fixed with an EM-fixative solution of cold 2.5% glutaraldehyde in 0.1 M phosphate buffer (pH 7.2) and 2% paraformaldehyde in 0.1 M phosphate or cacodylate buffer (pH 7.2) overnight and embedded in epoxy resin. Epoxy resin-embedded samples were loaded into capsules and allowed to form polymers (38 °C for 12 h and 60 °C for 48 h). Sliced sections were cut using an ultramicrotome (RMC MT-XL) and collected on a grid made of copper. Appropriate areas from thin sectioning were cut at 65 nm and stained with saturated 4% uranyl acetate and 4% lead citrate, and then examined with a transmission electron microscope (JEM-1400; Tokyo, Japan) at 80 kV. Mitochondrial length, damaged mitochondrial fraction per cell, and total mitochondrial number per cell were quantified manually. Seven cells per group were analyzed for two independent experiments.

### Intracellular and mitochondrial ROS level measurement

To detect intracellular ROS levels, cells were incubated with 1 μM dichlorofluorescein diacetate (Invitrogen) for 30 min and washed with warm phosphate-buffered saline (PBS). Mitochondria-specific ROS were detected using MitoSOX Red (5 μM for 15 min at 37 °C) following the manufacturer’s protocols (Invitrogen). For all mitochondrial assays, cells were treated with vehicle, 1 μM Aβ, or 500 ng/ml TSP-1 for 24 h, or pretreated with TSP-1 for 1 h and co-incubated with either 1 μM Aβ or DMSO for the next 23 h. Fluorescent signals were captured using a fluorescence microscope (Olympus), and >100 cells were analyzed for each group.

### Measurements of OCR

A Seahorse Bioscience XF24–3 extracellular flux analyzer (Seahorse Bioscience) was used to detect OCR when glucose, l-glutamine, and sodium pyruvate (Sigma-Aldrich) were added to Seahorse XF base medium (Seahorse Bioscience) surrounding HT22 cells. Cells were cultured in XF24 V7 cell culture microplates according to the manufacturer’s instructions. Briefly, OCR measurements were performed after equilibration of calibration medium (Seahorse Bioscience). Every 4 min, the O_2_ concentration was measured. OCR was recorded as a unit of pmol/min. Baseline rates were first measured. Then, preloaded oligomycin (2 μM; Seahorse Bioscience), FCCP (2 μM; Seahorse Bioscience), rotenone (0.5 μM: Seahorse Bioscience), and antimycin A (0.5 μM; Seahorse Bioscience) were injected in a consecutive order into each well of the cell plate in a calibration chamber during the Seahorse assay. After mixing the injected drugs and assay medium for 2 min, OCR was measured four to six times. The averages of five baseline rates and four to five test rates were used for data analyses.

### Live and dead cell assay

#### Calcein-AM assay

To measure cell viability, the calcein-AM assay was performed. Briefly, 5 × 10^3^ cells were incubated for 24 h after seeding in a 96-well plate, and then treated with Aβ and/or Tsp-1 for 24 h. Calcein-AM reagent (C3099, Molecular Probes, Invitrogen) in phenol red-free media (1 M) was added, incubated for 1 h at 37 °C, and washed three times with PBS. Fluorescence was measured at excitation and emission wavelengths (ex/em) of 485 nm/530 nm on a fluorescence plate reader (Infinite M200 Pro; TECAN, Mannendorf, Switzerland).

#### MTT assay

For measuring the activities of mitochondrial enzymes, MTT assays were performed^37^. Briefly, 2.5 mg/ml MTT (M2003; Sigma-Aldrich) in phenol red-free medium was added to the cells and incubated for 2 h at 37 °C, followed by the aspiration of the MTT solution, and the addition of isopropanol, which was incubated at 37 °C for 1 h to dissolve formazan crystals. Absorbance was measured at 540 nm. Experiments were repeated at least three times independently, and data were expressed as a percentage of the control cell.

#### TUNEL assay

The TUNEL assay was performed with a DeadEnd Colorimetric TUNEL System (Promega, Madison, WI, USA) according to the manufacturer’s instructions in rat primary hippocampal neurons.

For all cell viability and cell death assays, cells were treated with vehicle, 1 μM Aβ, or 500 ng/ml of TSP-1 for 24 h, or pretreated with TSP-1 for 1 h and co-incubated with 1 μM Aβ or DMSO for next 23 h.

### Intracellular calcium measurement

To measure intracellular calcium levels, Fluo-4 Direct™ Calcium Assay (Thermo Fischer Scientific) was performed following the manufacturer’s protocols. Briefly, 50 μl of the 2× Fluo-4 Direct™ calcium reagent loading solution was added to each well of a 96-well plate containing 50 μl of growth medium. The plate was then incubated at 37 °C for 30 min, followed by an additional 30 min incubation period at room temperature. Fluorescent signals were then measured using a fluorescence microscope. The intensity of the results was analyzed with the Image J program for three independent experiments.

### Data analysis and statistics

For western blot analysis, protein levels were normalized to pan forms or housekeeping proteins, such as β-actin or GAPDH. All data were expressed as means ± SEM). Statistical analysis was performed using GraphPad Prism 5. The data were analyzed by one-way analysis of variance with post hoc test (*p < 0.05, **p < 0.01, and ***p < 0.001).

## Electronic supplementary material

Supporting information
