# Supplementary material for: Thrombospondin-1 protects against Aβ-induced mitochondrial fragmentation and dysfunction in hippocampal cells
Source: Cell Death Discov. 2018 Feb 20;4:31. doi: 10.1038/s41420-017-0023-4 (PMC5841271; doi:10.1038/s41420-017-0023-4)
Supplement: Supplementary file 1 — Supporting information [file 41420_2017_23_MOESM1_ESM.docx]

**Supporting Information**

**
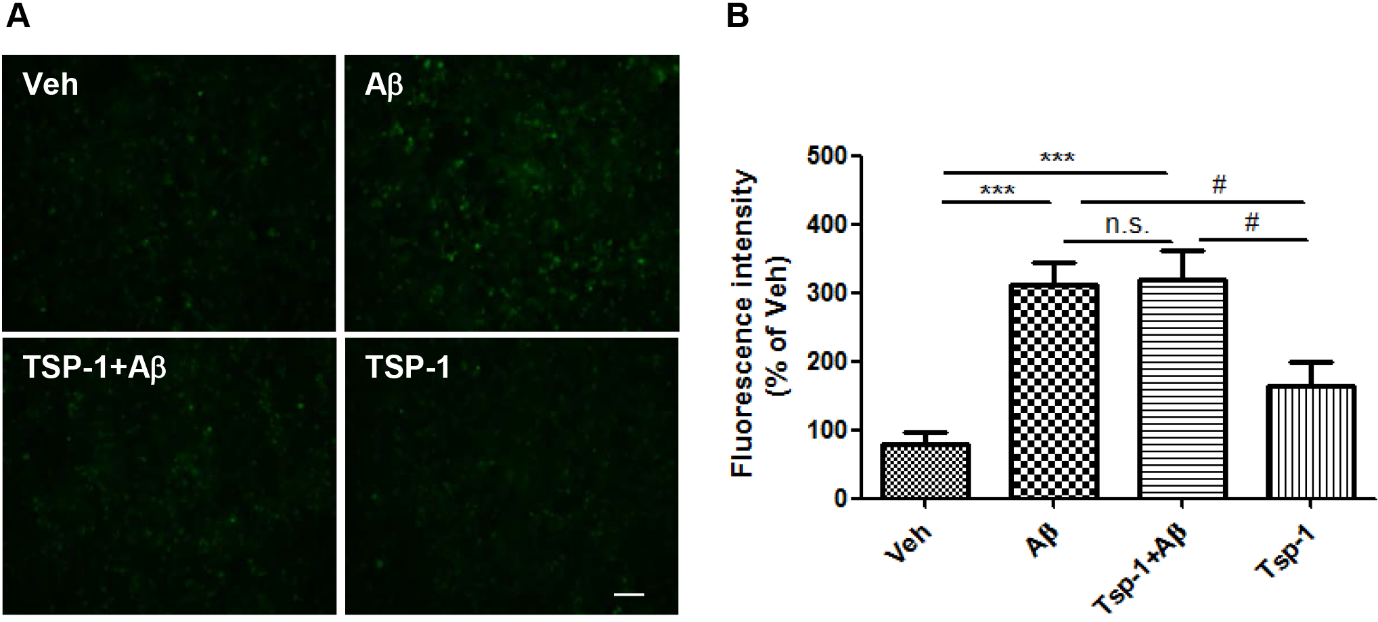
**

**Supplementary Figure 1: TSP-1 does not rescue elevated Ca^2+^ level in Aβ treated HT22 cells.** (a) To detect intracellular Ca^2+^ ion level, a fluo-4 assay was performed. (b) Despite the presence of TSP-1, an Aβ-induced intracellular Ca^2+^ increment was still observed in TSP-1 along with Aβ treated HT22 cell group. Data are presented as mean ± S.E.M. ***p < 0.001 vs. vehicle (DMSO) treated cells; #p < 0.05 vs. TSP-1 treated cells; n.s. means statistically nonsignificant. Scale bar, 50 μm.

**
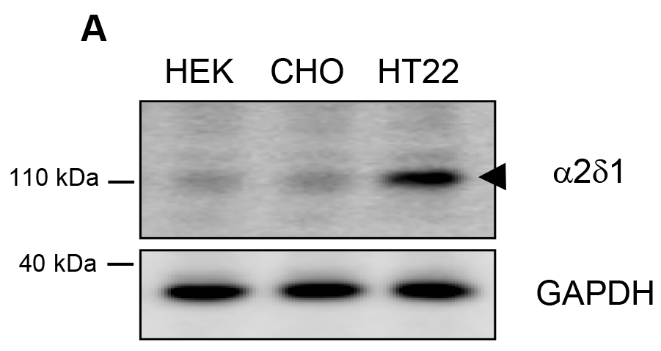
**

**Supplementary Figure 2: α2δ1 receptor is expressed in HT22 cells.** (a) α2δ1 receptor protein is highly expressed in HT22 cells compared to HEK or CHO cells.

**
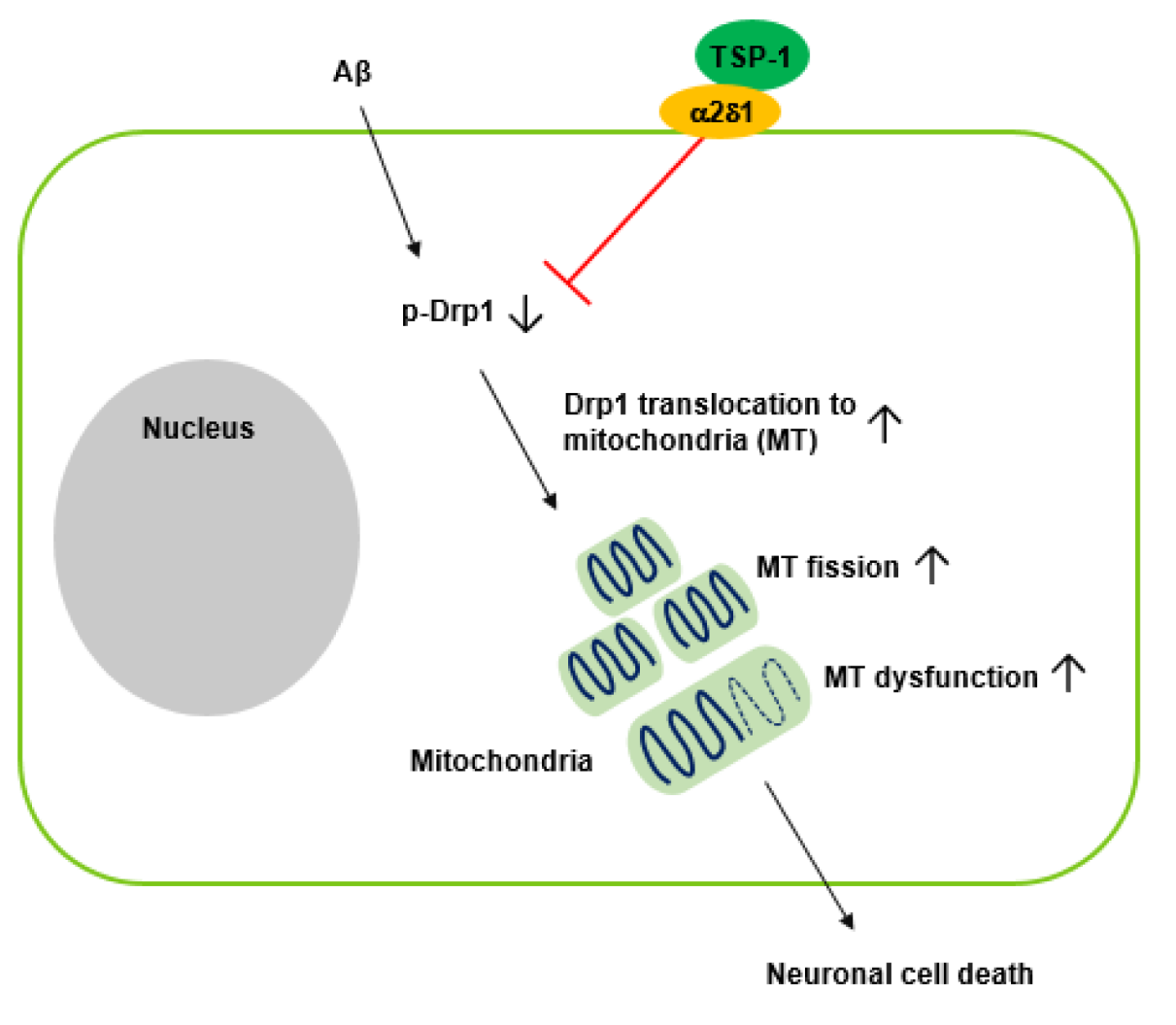
**

**Supplementary Figure 3: Schematic diagram of TSP-1 study.** In the presence of Aβ, the ratio of p-Drp1 (Ser 637) to Drp1 which is crucial for mitochondrial fission/fusion dynamics was decreased. As a result, massive mitochondrial fragmentation occurred by decreasing the ratio of p-Drp1 (Ser 637) to Drp1. An imbalance in mitochondrial dynamics induces mitochondrial dysfunction, disrupts mitochondrial quality control and finally contributes to neuronal cell death. In this study, we found that TSP-1 plays an important role in maintaining mitochondrial dynamics, function and even neuronal cell viability by maintaining the level of p-Drp1. In addition, this protective role of TSP-1 may be exerted mainly via the interaction of TSP-1 and its neuronal receptor, α2δ1.
